# Requirements of Postnatal proBDNF in the Hippocampus for Spatial Memory Consolidation and Neural Function

**DOI:** 10.3389/fcell.2021.678182

**Published:** 2021-07-15

**Authors:** Wei Sun, Hong Cheng, Yang Yang, Dongxin Tang, Xiaolian Li, Lei An

**Affiliations:** ^1^Behavioral Neuroscience Laboratory, The First Affiliated Hospital of Guizhou University of Traditional Chinese Medicine, Guiyang, China; ^2^Department of Pediatric, The First Affiliated Hospital, Guizhou University of Traditional Chinese Medicine, Guiyang, China; ^3^Department of Neurology, Guizhou University of Traditional Chinese Medicine, Guiyang, China; ^4^Department of Neurology, Jinan Geriatric Hospital, Jinan, China; ^5^Department of Physiology, University of Saskatchewan, Saskatoon, SK, Canada

**Keywords:** hippocampus, long-term depression, memory consolidation, NMDA receptors, proBDNF

## Abstract

Mature brain-derived neurotrophic factor (BDNF) and its downstream signaling pathways have been implicated in regulating postnatal development and functioning of rodent brain. However, the biological role of its precursor pro-brain-derived neurotrophic factor (proBDNF) in the postnatal brain remains unknown. The expression of hippocampal proBDNF was blocked in postnatal weeks, and multiple behavioral tests, Western blot and morphological techniques, and neural recordings were employed to investigate how proBDNF played a role in spatial cognition in adults. The peak expression and its crucial effects were found in the fourth but not in the second or eighth postnatal week. Blocking proBDNF expression disrupted spatial memory consolidation rather than learning or memory retrieval. Structurally, blocking proBDNF led to the reduction in spine density and proportion of mature spines. Although blocking proBDNF did not affect N-methyl-D-aspartate (NMDA) receptor (NMDAR) and α-amino-3-hydroxy-5-methyl-4-isoxazolepropionic acid receptor (AMPAR) subunits, the learning-induced phosphorylation of the GluN2B subunit level declined significantly. Functionally, paired-pulse facilitation, post-low-frequency stimulation (LFS) transiently enhanced depression, and GluN2B-dependent short-lasting long-term depression in the Schaffer collateral-CA1 pathway were weakened. The firing rate of pyramidal neurons was significantly suppressed around the target region during the memory test. Furthermore, the activation of GluN2B-mediated signaling could effectively facilitate neural function and mitigate memory impairment. The findings were consistent with the hypothesis that postnatal proBDNF played an essential role in synaptic and cognitive functions.

## Introduction

Mature brain-derived neurotrophic factor (mBDNF) plays an important role in neural circuit formation (Fernandes et al., 2015; Li et al., 2019), which is a critical step in aiding in hippocampus (HPC)-dependent memory in adolescents and adults (Lu et al., 2014; Itoh et al., 2016). Like many other neurotrophins, mBDNF is initially produced as a longer precursor molecule, pro-brain-derived neurotrophic factor (proBDNF), which elicits an opposing response to that of mBDNF (Lu et al., 2005; Deinhardt and Chao, 2014). For example, in contrast to the role of mBDNF in cell survival and memory formation, proBDNF can bind to p75^*NTR*^, induce apoptosis (Je et al., 2012; Sun et al., 2012) and axonal retraction (Yang F. et al., 2009), and inhibit neuronal migration (Xu et al., 2011). Hence, the interest has grown in understanding the underlying mechanism and roles of neurotrophins in synaptic competition and elimination during neural circuit formation (Yang F. et al., 2009; Je et al., 2012; Yang et al., 2014). Although the structural and functional roles of perinatal mBDNF in cognitive processing are defined (Lu et al., 2014), the potential roles of proBDNF are still unclear.

Research studies have shown that N-methyl-D-aspartate (NMDA) receptors (NMDARs) play important roles in synaptic plasticity, brain development, and learning and memory (Bannerman et al., 2014) and are also involved in BDNF-dependent cognitive development (Lu et al., 2014; Nakai et al., 2014; Itoh et al., 2016). The downregulation of mBDNF reduces, and exogenous mBDNF enhances NMDAR-mediated neural responses (Itoh et al., 2016). The activation of the cAMP-dependent protein kinase (PKA)/cAMP response-element binding protein (CREB) pathway by glutamate *via* the stimulation of NMDARs is essential for the effects of mBDNF on dendritic development and the formation of neural circuits during postnatal development (Finsterwald et al., 2010) by the selective strengthening of necessary synapses in an activity-dependent manner (Lu et al., 2005; Choo et al., 2017). Treatment of rats with ketamine, an NMDA-channel antagonist, caused a significant increase in CREB and mBDNF protein levels in the HPC, as well as PKA phosphorylation levels (Reus et al., 2011). More importantly, experiments conducted in BDNF heterozygous animals demonstrated that the subunit composition of NMDARs in the HPC was altered (Klug et al., 2012). Different effects were observed in dorsal hippocampal regions involved in learning and memory and ventral regions involved in fear and anxiety-like behavior. Intriguingly, both mBDNF and proBDNF are secreted in adulthood, but the highest levels of proBDNF are observed perinatally (Yang J. et al., 2009). The prenatal proBDNF requirement is impacted by neuronal depolarization (Yang J. et al., 2009), which can control the BDNF-induced expression of NMDAR subunits at the transcriptional level (Suzuki et al., 2005). Moreover, proBDNF negatively regulates neural remodeling by selectively facilitating NMDAR-dependent neurotransmission (Yang et al., 2014) and neural activity (Sun et al., 2019). Therefore, NMDARs may be important mediators of proBDNF-induced defects in neurodevelopment and neurocognition.

To address the aforementioned issues, the variations in the expression of hippocampal proBDNF (at different periods from birth to adulthood) were tested, and then the effects of blocking proBDNF at its peak expression on spatial learning and memory of adult rats were assessed. Using a combination of morphological, Western blot and pharmacological methods, this study attempted to identify the role of proBDNF in spine development and the expression and phosphorylation of the subunits of glutamatergic receptors [including α-amino-3-hydroxy-5-methyl-4-isoxazolepropionic acid receptors (AMPARs) and NMDARs]. Meanwhile, the role of proBDNF in the synaptic function of the Schaffer collateral-CA1 pathway and the neural correlates of spatial behaviors were also assessed. To further confirm the findings, the pharmacological tools were employed to mitigate proBDNF-mediated deficits in cognitive and neural functions. These findings might help further understand the mechanisms by which proBDNF exerted its effects on synaptic and cognitive functions.

## Materials and Methods

### Subjects

Wistar rats (Beijing Research Center for Experimental Animals, China) were maintained on a 12-h light/dark cycle (lights on at 7 a.m.) at constant temperature (21 ± 2°C) and humidity (45 ± 5%). All tests were conducted during the light period (between 2 p.m. and 5 p.m.). Animals had *ad libitum* access to food and water unless food was restricted prior to the training of lever press tests. During behavioral tasks, rats were maintained at ∼85% of free feeding weight, which was compared with a standard growth curve (Donaldson, 1924). All procedures were in accordance with the Care and Use of Animals Committee of Guizhou University of Traditional Chinese Medicine (SCXK-2013-0020).

The day of birth was designated as postnatal day (PD) 0, and pups were weaned on PD21. A total of 418 male offspring from an average of 84 litters were randomly assigned to one of six groups: (Fernandes et al., 2015) anti-proBDNF (second week), (Choo et al., 2017) anti-proBDNF (fourth week), and (Je et al., 2012) anti-proBDNF (eighth week) groups received bilateral infusion of rabbit polyclonal anti-proBDNF antibody (Lim et al., 2015; Luo et al., 2016) in the CA1 region of the HPC throughout the entire second postnatal week (PD2w, from PD8 to PD14), fourth postnatal week (PD4w, from PD22 to PD28), and eighth postnatal week (PD8w, from PD50 to PD56), respectively; (Li et al., 2019) control group was treated with the same volume of the vehicle (artificial cerebrospinal fluid, ACSF) throughout the whole PD2w (Con@2w), PD4w (Con@4w), and PD8w (Con@8w); (Itoh et al., 2016) Anti+TBOA group, which received infusion of anti-proBDNF antibody during the postnatal weeks, was bilaterally infused with DL-threo-β-benzyloxyaspartate (DL-TBOA) 0.5 or 2.5 h before spatial training [Anti+TBOA0.5(a) or Anti+TBOA2.5(a)], immediately following behavioral training [Anti+TBOA(b)] or 0.5 h before probe test [Anti+TBOA(c)]; and (Lu et al., 2014) control group, which received infusion of ACSF during the postnatal weeks, was bilaterally infused with DL-TBOA 0.5 h before spatial training [TBOA(a)], immediately following behavioral training [TBOA(b)] or 0.5 h before probe test [TBOA(c)]; (Lu et al., 2005) naive group was reared as the control group without the treatment. Eight-week-old (PD56) adult rats were used for this study unless specific evaluation was required. Anti-proBDNF antibody was purchased from Alomone Labs, Ltd. (Jerusalem, Israel; Cat. No. ANT-006). DL-TBOA was purchased from Tocris Cookson (Ellisville, MO, United States). ACSF was purchased from Beijing Leagene Biotechnology, Ltd. (Beijing, China).

Here, DL-TBOA was used to block glutamate transporters and increase extracellular glutamate levels, which in turn could activate the extrasynaptic GluN2B-NMDA receptor (Massey et al., 2004; Yang et al., 2005). Given that sex differences in BDNF signaling have been reported extensively (Kellogg et al., 2000; Wu et al., 2013; Luoni et al., 2016) and some molecular mechanisms in memory formation are also known to be sex-specific (Mizuno and Giese, 2010; Sundermann et al., 2016), only male rats were selected for the current study. Additionally, to exclude the possibility of accumulative effects of the drugs, separated groups were assigned in each behavioral experiment.

### Surgery and Microinjection

Rats were anesthetized with isoflurane and placed in a stereotaxic frame (SN-3; Narishige, Japan) for surgery. Guide cannulae (22 gauge; Plastics One Inc., Roanoke, VA, United States) were bilaterally inserted above the CA1 region of the HPC (for PD2w: AP: −3.3 mm, ML: ±2.1 mm, DV: 2.4–2.6 mm; for PD4w: AP: −3.3 mm, ML: ± 2.3 mm, DV: 2.6–2.8 mm; for PD8w: AP: −3.3 mm, ML: ± 2.3 mm, DV: 2.6–2.9 mm). A stainless-steel stylet (30 gauge, 10 mm; Plastics One Inc.) was inserted into guide cannula to avoid obstruction. Rats were given at least 1 week to recover.

Infusions were achieved by inserting 30-gauge needles (10 mm; Small Parts Inc., Logansport, IN, United States) connected through PE-50 tube into a microsyringe pump (Harvard Apparatus, Holliston, MA, United States), extended 1.0 mm beyond the end of the cannulae. Needles were inserted into both cannulae, and then anti-proBDNF antibody (10 μg/μl), DL-TBOA (2.0 ng/μl), Ro25-6981 (2.0 ng/μl), or ACSF (vehicle) was infused into the HPC area (0.5 μl/min/side for 2 min) 30 min before testing began. The dose and route of administration were selected based on the results of the previous studies, which indicate the efficacy of anti-proBDNF antibody (Bai et al., 2016; Sun et al., 2018a, 2019). To testify whether the TBOA infusion 0.5 h before the training could still affect memory consolidation, DL-TBOA infusion was conducted 2.5 h before the training. The needles were left for an additional 3–5 min to allow the diffusion. Specifically, anti-proBDNF antibody was applied twice a day in a 12-h interval (at 9 a.m. and 9 p.m.) for 1 week. Drug treatments were counterbalanced across litters.

### Protein Preparations and Analysis

Rats were killed by overdose of urethane, and hippocampi were rapidly dissected and homogenized in ice-cold lysis buffer (pH 7.4) containing a cocktail of protein phosphatase and proteinase inhibitors (Sigma, MA, United States). The samples were centrifuged at 14,000 rpm for 15 min at 4°C, and the supernatant was collected. Protein concentrations were detected by the bicinchoninic acid (BCA) assay. Twenty micrograms (15 μl) of total protein per lane was resolved in 10–15% SDS-PAGE gels followed by electro-transferring to PVDF membranes (Pall, Pensacola, FL, United States). Non-specific binding of antibodies to membranes was probed with the primary antibody: mouse anti-proBDNF (1:500, Cat. No. sc-65514; Santa Cruz Biotechnology, Santa Cruz, CA, United States), mouse anti-mBDNF (1:500, Cat. No. mab248; R&D Systems, Minneapolis, MN, United States), rabbit anti-p75^*NTR*^ (1:1,000, Cat. No. AB1554; Chemicon, CA, United States), rabbit anti-GluA1 (1:1,000, Cat. No. AB1504; Chemicon, CA, United States), rabbit anti-phospho(serine-831)GluA1 (1:500, Cat. No. 04823; Upstate Biotechnology, MA, United States), rabbit anti-mGluA2/3 (1:1,000, Cat. No. AB1506; Chemicon, CA, United States), rabbit anti-phospho(serine-880)GluA2 (1:3,000, Cat. No. 07294; Upstate Biotechnology, MA, United States), rabbit anti-GluN2A (1:1,000, Cat. No. 07632; Millipore, MA, United States), rabbit anti-phospho(serine-1232)GluN2A (1:1,000, Cat. No. crb2005001e; Cambridge Research Biochemicals, Billingham, United Kingdom), mouse anti-GluN2B antibody (1:1,000, Cat. No. 06600; Millipore, MA, United States), rabbit anti-phospho(serine-1303)GluN2B (1:1,000, Cat. No. ab81271; Abcam, Cambridge, United Kingdom), and mouse anti-β-actin (1:20,000, Cat. No. A5316; Sigma, MA, United States) overnight at 4°C. Mouse anti-β-actin was used as an internal control. Each band was normalized to the corresponding β-actin band. After further incubation in horseradish-peroxidase (HRP)-conjugated secondary goat anti-mouse or anti-rabbit IgG (1:1,000) (Southern Biotechnology Associates, AL, United States) for 2 h at room temperature, immunoreactivity was detected by ECL Western Blotting Detection Kit (CWBIO, China). The intensity of each band was measured by densitometry using Quantity One software (Bio-Rad Laboratories, Hercules, CA, United States). The learning-induced expression level was normalized by the expression of the naive group.

### Locomotion and Anxiety-Like Behavior in the Open Field Task

Locomotor activity was assessed in a 5-min open field, which consisted of a 91.5 × 91.5 × 61 cm Perspex box with dark walls, as described previously (Mueller et al., 2010; Peters et al., 2010). The field was divided into a peripheral region (within 15.25 cm of the walls) and central region (61 × 61 cm) of approximately equal area. The distance traveled and the time spent within the peripheral/central region were recorded using VersaMax Activity Monitoring System (AccuScan Instruments, Columbus, OH, United States).

### Motivation Test

Rats were trained to lever press for food pellets in standard operant conditioning chambers located inside sound-attenuating boxes (Med Associates, St. Albans, VT, United States). The chambers contained two retractable levers located on either side of a central food trough. As in the previous studies (Paterson et al., 2005; Sun et al., 2020, 2021b), rats were trained daily in 30-min sessions with one of two levers extended randomly when the cue light above the lever was on. The training started with continuous reinforcement. Rats were initially trained on a fixed-ratio (FR)-1 schedule (one lever-press response) with both levers reinforced, followed by the sequence FR-15, FR-30, and finally FR-60 schedule sessions. Rats were tested in a 30-min session till they reached 10 presses per min on FR-60.

### MWM Test

A 150-cm-diameter circular pool was filled with water opacified with nontoxic black ink and kept at 25 ± 1°C. The tank was divided into four equal quadrants that were denominated clockwise I, II, III, and IV. A clear 10-cm-diameter platform was positioned in the center of quadrant III with its surface 2 cm below the water surface. The pool was surrounded by blue curtains with clearly distinctive cues. Movements were monitored by a tracking system (Ethovision 2.0; Noldus, Wageningen, Netherlands).

The test was divided into the training phase on day 1 and the probe phase, which was performed 24 h or immediately after training. During the training phase, each rat was trained for eight trials (30 s intertrial interval) to find the platform. The order of starting points was set pseudorandomly (II, I, III, IV, III, I, IV, II) but was the same for all animals. Rats that failed to find the platform within 60 s were guided and remained on it for 20 s. The escape latency of each trial was collected and calculated. During the probe phase, the platform was taken out, and rats were released from a novel drop point (between starting points I and II) and swam for 60 s. From the tracked swimming traces, a path proximity score was calculated by measuring the distance (cm) between the rat’s position and the platform location (Maei et al., 2009; Tomas Pereira and Burwell, 2015; Kapadia et al., 2016). A distance measure was made 10 times per second and averaged across the probe test.

The long-term memory process can be generally divided into distinct stages: learning (acquisition), consolidation, and retrieval (Wang et al., 2006). Extensive studies have confirmed that the newly formed memories were susceptible to a variety of post-learning (minutes to half hour) manipulations, such as electroconvulsive shock, protein synthesis inhibitor, or hypothermia treatment (McGaugh, 2000; Kandel, 2001). Moreover, the disruptive effects of these post-learning manipulations decrease as the time interval between the acquisition and the intervention increases (Dudai et al., 2015). Intensive research in the past several decades suggests that this type of memory consolidation, occurring within minutes to hours after initial learning, may reflect the ongoing changes in the intracellular signaling pathways and new protein synthesis and gene expression by which subsequent modifications in synaptic properties and structures are produced (Izquierdo et al., 2006; Nadel et al., 2012). Regarding the conversion of short-term memory into long-term memory, in the Morris water maze (MWM) task, the memory acquisition is during the training phase on the first day. After memory acquisition, the memory is consolidating and will be assessed on the probe test on the second day. This eight-trial training, which can quickly be learned by rodents in the previous studies (de Quervain et al., 1998; Wong et al., 2007; Dong et al., 2013), has the advantage of clearly delineating the acquisition and memory consolidation phases (Ge et al., 2010; An and Sun, 2018).

### Single-Unit Recording

One week before behavioral test, electrode implantation was conducted using previously reported procedures (Sun et al., 2018b, 2021, 2021a). Briefly, rats were anesthetized with isoflurane and prepared for surgery. Impedance-measured (200–600 kΩ) microelectrodes were arrayed into a 4 × 8 matrix using 25-μm-diameter tungsten wires (California Fine Wires, Grover Beach, CA, United States) in a 35-gauge silica tube (World Precision Instruments, Sarasota, FL, United States). A cannula was attached to a silica tube. The proximal open end of the cannula was parallel to electrode tips. They were chronically implanted, and the left or right hemisphere was implanted randomly but counterbalanced between rats. A stainless-steel wire was used as ground electrode, and the electrode was fastened to the cranium by dental acrylic with skull screws.

Data were acquired on a Digital Cheetah system (Cheetah software; Neuralynx Inc., Bozeman, MT, United States). Unit signals were recorded *via* an HS-36-unit gain headstage (Neuralynx Inc.) mounted on the animal’s head by means of lightweight cabling that passed through a commutator (Neuralynx Inc.). Unit activity was amplified (1,000–10,000 times) and sampled at 32 kHz and 600–6,000 Hz band-pass filters. The firing rates during the probe test were collected. The rats’ behavior was monitored by a digital ceiling camera (Neuralynx Inc.), and the CCD camera’s signal was fed to a frame grabber (sampling rate, 1 MHz) with the experimental time superimposed for offline analysis.

Spike sorting was performed with offline Neuralynx’s software (SpikeSort 3D), using a combination of KlustaKwik, followed by manual adjustment of the clusters (Klusters software package). Briefly, multiple parameters were used to determine the clusters with the most often used combination of spike height, trough, and energy, associated with the waveforms (Hernandez et al., 2013; An et al., 2018). As in the previous studies (Stark et al., 2014; Sun et al., 2018b), units were graded for quality and classified as pyramidal neurons and fast-spiking (FS) interneurons.

### Synaptic Plasticity at the Schaffer Collateral-CA1 Pathway

*In vivo* field excitatory postsynaptic potentials (fEPSPs) in the pyramidal layer of the hippocampal CA1 region were recorded as previously explained in the Materials and methods section (An and Sun, 2017, 2018; An et al., 2019). Briefly, rats were anesthetized with isoflurane and placed in a stereotaxic frame for surgery (SN-3; Narishige, Japan). Core body temperature was monitored throughout the experiment, and a heating pad was used to maintain the temperature of the animals at 36.5 ± 0.5°C. The scalp was opened, and small holes were drilled in the skull using a trephine for the monopolar recording (insulated platinum iridium wire; AM Systems; AP: −3.3 mm, ML: ± 2.3 mm, DV: 2.6–2.8 mm) and tungsten bipolar stimulating electrodes (FHC; ME; hippocampal Schaffer collaterals region; AP: −4.0 mm, ML: ± 3.3 mm, DV: −2.2 to −3.0 mm). The head side of each rat was chosen randomly but counterbalance among groups. After the electrodes were lowered and located properly in desired positions, input/output (I/O) curves and paired-pulse facilitation (PPF) were assessed. The frequency of test pulse recording ranged from 30 to 60 s. A baseline recording was re-established for approximately 5–10 min following the completion of each recording. Low-frequency stimulation (LFS) (900 pulses of 1 Hz) was delivered to induce long-term depression (LTD). The stimuli were delivered every minute at an intensity that evoked a response of 60–70% of the maximum response, which was obtained from the I/O recording. Since LTD should last for at least hours, the expression of LTD in the current study can only be defined as a short-lasting long-term depression (SL-LTD). Initial data measurement was performed in Clampfit 9.0 (Molecular Devices, Sunnyvale, CA, United States). The fEPSPs slope was used to measure synaptic efficacy. The average amplitudes during the baseline period were normalized to 100%, and the relative amplitudes at every point were normalized relative to the baseline period. The average amplitude between 41 and 60 min after the completion of the LFS was used to analyze.

### Spine Density Analysis

Immediately following the probe test (24 h after the training stage), rats were anesthetized by an intraperitoneal injection of sodium pentobarbital (80 mg/kg). The brains were removed without perfusion, rinsed in phosphate-buffered saline (PBS), and stained using the Golgi–Cox method, in accordance with the manufacturer’s instructions (Rapid GolgiStain; FD Neurotechnologies, United States). Briefly, brain tissues were immersed in the impregnation solution made by mixing equal volumes of solutions A and B and stored at room temperature for 10 days in the dark. The brains were then transferred into solution C and stored at 4°C in the dark for 5 days. Sections were cut on a vibratome and mounted on gelatin-coated slides with solution C for natural drying at room temperature for 2 days. Brain sections (50 μm) that could be clearly evaluated and containing 50–150 μm of secondary dendrites from each imaged soma were selected (Yang C. et al., 2015; Li et al., 2017). Three CA1 pyramidal neurons per section and three sections per animal were analyzed. Each rat was treated as an independent sample. For spine categorization, the following criteria were used (Li et al., 2017): (Fernandes et al., 2015) mushroom: spine head diameter was ≥1.5 × spine neck diameter; (Choo et al., 2017) stubby: spine head and spine neck were approximately of the same width, and spine length was not significantly longer than head diameter; and (Je et al., 2012) thin: spine head and spine neck were approximately of the same width, and spine length was 2.5 times longer than spine head width. Spine densities were calculated as the mean number of spines per micrometer dendrite.

### Statistical Analysis

**FIGURE 1:**
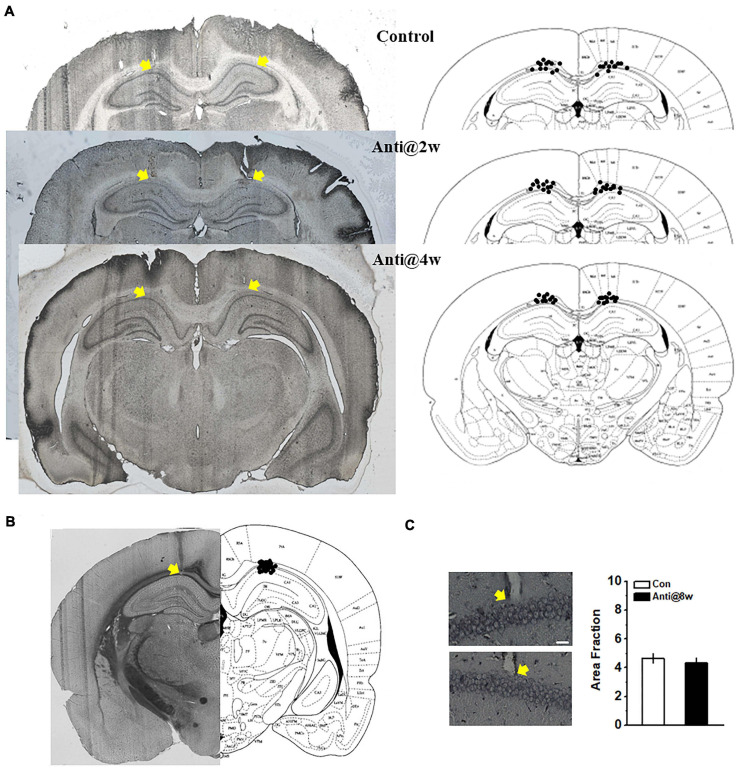
Schematic representations of the cannulae and electrode placements and morphological alterations in the CA1 region. **(A)** Histological (left) and schematic (right) representations of the cannula placements. The control group infused with ACSF throughout the whole PD4w; the Anti@2w and Anti@4w groups were infused with anti-proBDNF antibody throughout the whole second and fourth postnatal weeks, respectively. The yellow arrows indicated the top of the cannulae. **(B)** Histological and schematic representations of electrode placements. **(C)** Following the open field test, infusion-induced neuronal damage was assessed by Silver staining (see Supplementary Methods). The white scale bar presented at the bottom of the photomicrograph indicated 25 μm. The yellow arrows indicated the electrode tips. There was no statistical difference in the quantification of neurodegeneration in CA1 neurons between the control (top) and anti-proBDNF (bottom) groups. The anti-proBDNF group was infused with anti-proBDNF antibody throughout the whole fourth postnatal week. The control group was treated with the same volume of the vehicle (ACSF) throughout the whole the fourth postnatal week. The treatment was conducted twice a day in a 12-h interval. *n* = 6 for each group.

To confirm the infusion and recording sites, electrolytic lesions were created by applying direct current (10 mA, 10 s). The infusion sites (see Figure 1A) and electrode placements (see Figure 1B) were identified with the aid of The Rat Brain in Stereotaxic Coordinates (1997, third edition). Only data obtained from rats with correctly inserted needles and probes were included in statistical analysis (see Figure 5I – top and Figure 5I – bottom).

**FIGURE 2:**
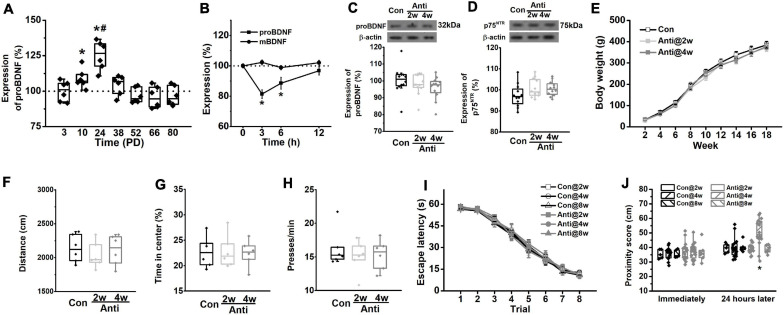
Blockage of proBDNF expression during the postnatal period induces spatial learning impairments. **(A)** The level of proBDNF in the hippocampus. (*p < 0.05, vs. 100%; #*p* < 0.05, vs. PD10). *n* = 6 per time point. **(B)** The expression of proBDNF in the hippocampus immediately, 3, 6, and 12 h after anti-proBDNF antibody infusion. *n* = 6 per group. (*p < 0.05, vs. matched mBDNF). **(C)** The proBDNF level and **(D)** the p75NTR level at PD56 were detected in rats, which were infused with anti-proBDNF antibody throughout the second postnatal week (Anti2w) and the fourth postnatal week (Anti4w), respectively. *n* = 12 per group. **(E)** Bodyweight changes from PD14 to PD126. **(F)** Travel distance. **(G)** The percentage of time spent in the center of the apparatus in the open field test. *n* = 6 per group. **(H)** Press time per min in the motivation test. *n* = 6 per group. **(I)** Escape latency. **(J)** The swim proximity score during the MWM task. Note that rats in the Con@8w and Anti@8w groups were tested at PD12w, whereas rats in other groups were tested at PD8w. (*p < 0.05, vs. Anti@2w, Anti@8w, or Con). *n* = 5 for the Con@2w group, *n* = 5 for the Con@8w group, *n* = 6 for the Anti@8w group, and *n* = 16 for other each group.

**FIGURE 3:**
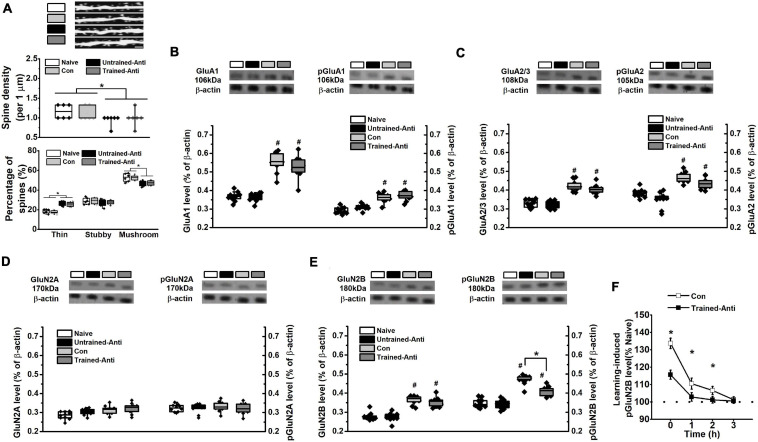
Blocking proBDNF reduces spine number and learning-related GluN2B expression. The samples from rats that performed spatial training in the MWM task were collected immediately following the training phase and selected for detecting spine density and the expression of glutamatergic receptor subunits. **(A)** Spine alteration in naive, untrained-antiproBDNF, trained control, and trained-antiproBDNF rats (top). Quantification of spine density (middle) and the proportion of spine (bottom). Scale bars, 5 μm. *n* = 6 per group. Sample images were projected at minimal intensity and inverted, background was then subtracted, followed by brightness/contrast adjustment. The expression and phosphorylation of GluA1 **(B)** and the expression of GluG2/3 and the phosphorylation of GluA2 **(C)** of AMPAR subunits. *n* = 10 per group. The expression and phosphorylation of GluN2A **(D)** and GluN2B **(E)** of NMDAR subunits. (*p < 0.05, Con vs. Trained-Anti; #*p* < 0.05, vs. matched Naive and Untrained-Anti). *n* = 10 per group. Note that the data of dendritic spine and glutamatergic receptors tests were subjected to a two-way ANOVA in which training status (trained or not trained) and treatment (anti-proBDNF antibody, ACSF, or no treatment) were dependent variables. **(F)** The expression of pGluN2B immediately, 1, 2, and 3 h following spatial training. (*p < 0.05, Con vs. Trained-Anti). *n* = 10 per group.

**FIGURE 4:**
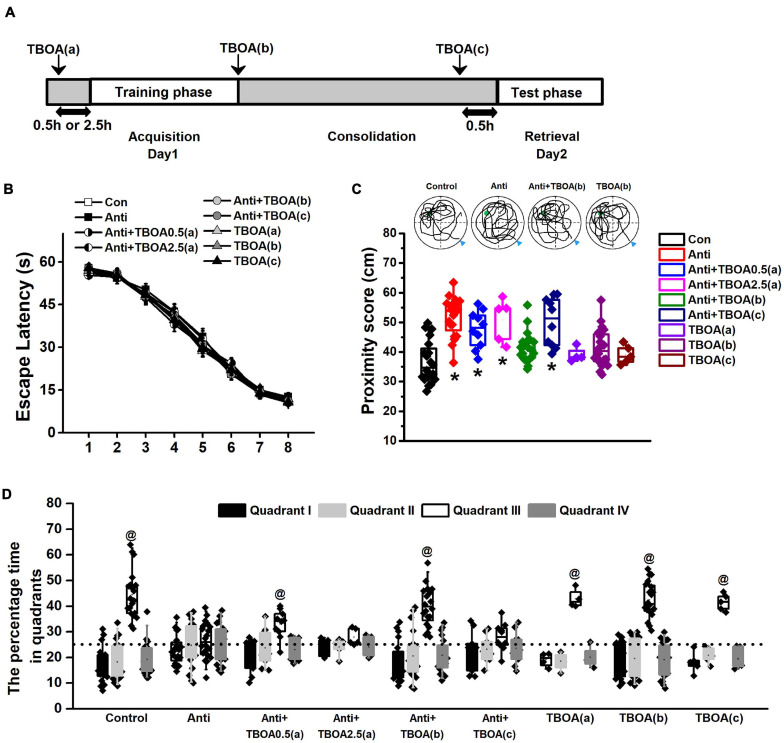
Activation of GluN2B can rescue memory consolidation induced by blocking postnatal proBDNF. The infusion of TBOA was conducted 0.5 (Anti+TBOA0.5(a)) or 2.5 h (Anti+TBOA2.5(a)) before spatial training (acquisition), immediately following training (consolidation; Anti+TBOA(b)), and 30 min prior to probe memory test (retrieval; Anti+TBOA(c)), respectively. **(A)** Schematic description of the experimental timeline. **(B)** Escape latency in the training phase and **(C)** the swim proximity score during the probe trial. Note the sample swimming traces demonstrating the swimming trajectories of the control, Anti+TBOA(b), and TBOA(b) groups rather than the Anti group superimposed on target quadrant. The triangle indicated the start point during probe trial. (*p < 0.05, vs. control, Anti+TBOA(b), TBOA(a), TBOA(b), or TBOA(c)). **(D)** The percentage of time spent in each quadrant during the probe test. @ *p* < 0.05, vs. other quadrants. Note the data from rats (control, Anti, Anti+TBOA(b), and TBOA(b) groups) used in the single-unit recording were included. *n* = 20 for the control, Anti, Anti+TBOA(b), and TBOA(b) groups, *n* = 10 for the Anti+TBOA0.5(a) and Anti+TBOA(c) groups, *n* = 5 for the Anti+TBOA2.5(a) group, *n* = 4 for the TBOA(a) group, and *n* = 5 for the TBOA(c) group.

**FIGURE 5:**
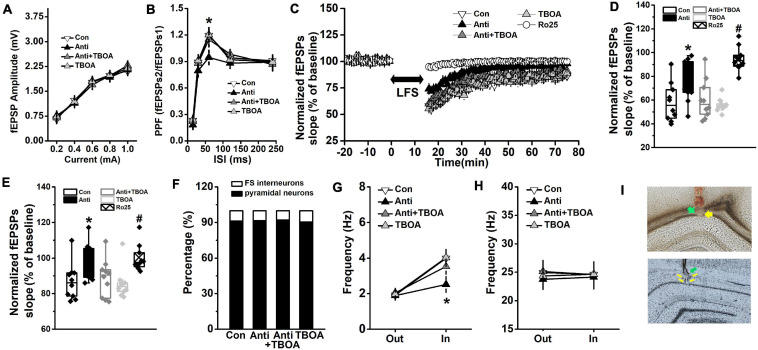
GluN2B-dependent neural function is enhanced by TBOA. The Anti group was bilaterally infused with anti-proBDNF antibody into the CA1 region throughout the whole PD4w, whereas the Con group received the same volume of ACSF. Eight-week-old rats were selected for detecting hippocampal synaptic function in the Schaffer collateral-CA1 pathway immediately following TBOA (Anti+TBOA and TBOA groups), Ro25 (Ro25 group), or ACSF (Con and Anti groups) injection. **(A)** Input–output curves of fEPSP slopes. **(B)** PPF, a form of short-term plasticity, was measured and expressed as the ratio of fEPSPs2 to fEPSPs1. (*p < 0.05, Anti vs. other groups). **(C)** Characteristic time courses of fEPSP slope. Arrow represented application of LFS. **(D)** The effects on PTD, which was determined as response 1 min after LFS. (*p < 0.05, Anti vs. other groups; #*p* < 0.05, Ro25 vs. other groups) **(E)** Time coursing changes in fEPSPs slope. Magnitude of SL-LTD was determined as responses in the last 20 min (between 56 and 75 min). (*p < 0.05, Anti vs. other groups; #*p* < 0.05, Ro25 vs. other groups). *n* = 10 per group. When rats were tested in the probe trial that was conducted 24 h following the last training trial, neural activity around the target platform area was recorded. TBOA (Anti+TBOA and TBOA groups) or ACSF (Con and Anti groups) infusion was conducted 30 min prior to the behavioral test. **(F)** The proportion of pyramidal neuron and FS interneuron. **(G)** Firing rate of pyramidal neurons out of target area (Out) and around the targeted platform (In) during the probe test. **(H)** Firing rate of FS interneuron out of target area (Out) and around the targeted platform (In) during the probe test. (*p < 0.05, Anti vs. other groups). *n* = 10 per group. **(I)** The histological representations of the recording sites during the fEPSPs (top) and neuronal activity (bottom) experiments. The green and yellow arrows indicate infusion site and recording site, respectively.

Data are expressed as mean ± SEM. All analyses were performed with Neuroexplorer, Matlab (MathWorks) and SPSS 17.0 software. The data of the training stage during the MWM task, bodyweight changes, I/O curve, and PPF were compared using repeated measures ANOVA. Student’s *t*-tests examined the data of histological observation, the expression of proBDNF during the postnatal period (Figure 2A), and the comparison of proBDNF and mBDNF levels to the 100% baseline level (Figure 2B). The percentage of time spent in quadrants (Figure 4D) was examined by Chi-square test. The data of Western blot tests, open field test, and lever-press test; the proximity score in the probe test (Figure 4C); and the normalized fEPSPs (Figures 5D,E) were examined by one-way ANOVA. A two-way ANOVA was employed to examine the data of the proximity score (Figure 2J), spine density, learning-induced pGluB2B level (Figure 3F), and neural firing frequency (Figures 5G,H). When the ANOVA reveals a significant main effect or interaction between main factors, data were further analyzed by Tukey’s *post hoc* test. For comparisons of the percentage of neurons, Pearson’s analyses were used. A *p* < 0.05 level of confidence was used in the analyses.

## Results

### Blocking the Expression of Hippocampal proBDNF During the Postnatal Period Impairs Spatial Memory but Not Learning Ability

Figure 2A shows the changes of proBDNF levels across development in the HPC of the un-manipulated rats. All data were normalized by the level at PD3. The expression rose significantly from PD3 to PD24 (one-way ANOVA, effect of time: *F*_(6,35)_ = 32.68, *p* < 0.001; *post hoc*, PD2w or PD4w vs. PD0w, both *p* < 0.05). The proBDNF levels peaked at PD24, which was significantly higher than that at PD10 (*p* < 0.05). To detect whether neutralizing proBDNF with its antibody would potentially interfere with the expression of endogenous protein and its proteolysis to mBDNF, we assessed the proBDNF and mBDNF levels following the antibody infusion (Figure 2B). Two-way ANOVA revealed significant time effect (*F*_(3,30)_ = 96.76, *p* < 0.001), significant treatment effect (*F*_(1,10)_ = 8.81, *p* < 0.01), and significant interaction effect between time and treatment (*F*_(3,30)_ = 32.63, *p* < 0.001). Tukey’s test showed that proBDNF levels were markedly lower than mBDNF levels 3 (*p <* 0.05) and 6 h (*p <* 0.05) following antibody infusions. The marked decline of proBDNF lasted for at least 6 h after the injection (*t*-test, 3 or 6 h vs. 100%, both *p* < 0.05), whereas the mBDNF level was not affected. Therefore, the observations following infusion reflected merely the proBDNF rather than the mBDNF effect. To confirm if infusion of anti-proBDNF antibody affects the level of proBDNF or its receptor, p75^*NTR*^, we assessed their levels at PD56. Hippocampal proBDNF was not disrupted by postnatal blockage at PD2w or PD4w (Figure 2C; one-way ANOVA, effect of treatment: *F*_(2,15)_ = 0.22, *p* > 0.05), neither did the p75^*NTR*^ (Figure 2D; effect of treatment: *F*_(2,15__)_ = 0.29, *p* > 0.05). We failed to find a statistical difference in bodyweight from PD2w to PD18w, either (Figure 2E; repeated-measures ANOVA, effect of time: *F*_(8_,_264__)_ = 0.51, *p* > 0.05; interaction effect between time and treatment: *F*_(__16__,264)_ = 0.27, *p* > 0.05). Furthermore, the effects of infusion on locomotion, anxiety-like behavior, and motivation were tested, whereas no statistical difference was found in the total travel distance (Figure 2F, one-way ANOVA, effect of treatment: *F*_(__2__,1__5__)_ = 0.23, *p* > 0.05) and the percentage of time spent in the center of the apparatus (Figure 2G, one-way ANOVA, effect of treatment: *F*_(2,15)_ = 0.29, *p* > 0.05) in the open field test, or the motivation behavior (Figure 2H, one-way ANOVA, effect of treatment: *F*_(2,15)_ = 0.26, *p* > 0.05). Blocking proBDNF at PD2w, PD4w, or PD8w did not disrupt spatial acquisition, as exhibited by a significantly decreased latency among groups in the training phase (Figure 2I, repeated-measures ANOVA, effect of trial: *F*_(__7,__406__)_ = 76.29, *p* < 0.001; effect of age: *F*_(2,61)_ = 0.68, *p* > 0.05; effect of treatment: *F*_(__1_,_62__)_ = 1.05, *p* > 0.05; interaction effect between age and treatment: *F*_(__2_,_124__)_ = 0.21, *p* > 0.05). Additionally, the mean time spent in thigmotaxis and floating during spatial training was comparable among groups (Supplementary Figure 1). However, infusion at PD4w, but not PD2w or PD8w, made rats away from target quadrant 1 day after acquisition training (Figure 2J, two-way ANOVA, effect of treatment: *F*_(__1_,_62__)_ = 18.71, *p* < 0.001, *post hoc*, Anti@4w vs. other groups, all *p* < 0.05; effect of age: *F*_(2,124)_ = 1.03, *p* > 0.05; effect of between treatment and age: *F*_(__2_,_124__)_ = 0.34, *p* > 0.05), but not immediately following training (effect of treatment: *F*_(__1_,_62__)_ = 0.26, *p* > 0.05). Additionally, our findings indicate that blocking proBDNF expression but not affecting p75^*NTR*^ expression or function by the infusion of anti-proBDNF antibody induces behavioral deficits in adults (Supplementary Figure 2). There was no statistical difference in area fraction between the Con and Anti@4w groups (Figure 1C, *t*-test, *t*_10_ = 0.0, *p* > 0.05). Our findings also ruled out the possibility that repeated infusions induced neuroinflammatory or neurodegeneration has contributed to the behavioral and physiological changes. Furthermore, our previous study found that exogenous proBDNF exerts pivotal effects on the use of cognitive strategies to facilitate the spatial learning process (An et al., 2018). Therefore, it remains possible that the deficit in memory consolidation was driven by a less precise learning strategy. However, blocking proBDNF during the postnatal period did not induce the learning strategy preference (Supplementary Figure 3). Together, the above results demonstrate the essential role of hippocampal proBDNF at PD4w in spatial memory function in adulthood. Therefore, we chose to block proBDNF activity at PD4w in the following experiments.

### Blocking Postnatal proBDNF Expression Decreases Spine Density and Learning-Induced Phosphorylated GluN2B-NMDA Receptor Subunit Level

At an early developmental stage, proBDNF is an important regulator of dendritic structure and synaptic plasticity. Crucially, endogenous proBDNF regulates learning-induced phosphorylation of glutamate receptors and spatial memory formation (Deinhardt and Chao, 2014; Yang et al., 2014; Shirayama et al., 2015; Sun et al., 2018a). Collectively, the spine density and the subunits of glutamate receptors were estimated during the memory formation period, which is generally believed to end within 0–3 h following the learning phase (Alonso et al., 2002; Slipczuk et al., 2009; Haynes et al., 2015). We failed to find learning-induced modifications in spine density (Figure 3A – middle, two-way ANOVA, *F*_(__1,22__)_ = 0.16, *p* > 0.05) or interaction effect between treatment and training (*F*_(1,22)_ = 0.09, *p* > 0.05), whereas two-way ANOVA revealed a significant anti-proBDNF antibody treatment effect (*F*_(1,22)_ = 17.27, *p* < 0.001). Furthermore, we classified spines into mushroom, stubby, and thin spines and found a significant anti-proBDNF antibody treatment effect (Figure 3A – bottom, two-way ANOVA, *F*_(1,22)_ = 20.31, *p <* 0.05) but no interaction effect between treatment and training (*F*_(1,22)_ = 0.13, *p* > 0.05) or training effect (*F*_(1,22)_ = 0.18, *p* > 0.05). Two-way ANOVA analysis indicated that a significant effect of training was found in GluA1 (Figure 3B – left, *F*_(1,38)_ = 16.55, *p <* 0.01), phosphorylated GluA1 (pGluA1, Figure 3B – right, *F*_(1,38)_ = 10.29, *p* < 0.01), GluA2/3 (Figure 3C – left, *F*_(1,38)_ = 13.37, *p* < 0.01), and phosphorylated GluA2 (pGluA2, Figure 3C – right, *F*_(1,38)_ = 15.83, *p* < 0.01), but not GluN2A (Figure 3D – left, *F*_(1,38)_ = 0.54, *p* > 0.05) or phosphorylated GluN2A (pGluN2A, Figure 3D – right, *F*_(1,38)_ = 0.47, *p* > 0.05). Meanwhile, statistical differences in pGluA1 expression were found between the Con and Naive groups (*p* < 0.05) and the Trained-Anti and Untrained-Anti groups (*p* < 0.05). There was no statistical effect of anti-proBDNF antibody infusion on GluA1 (*F*_(1,38)_ = 0.36, *p* > 0.05), pGluA1 (*F*_(1,__38__)_ = 0.57, *p* > 0.05), GluA2/3 (*F*_(1,__38__)_ = 0.62, *p* > 0.05), pGluA2 (*F*_(1,__38__)_ = 0.65, *p* > 0.05), GluN2A (*F*_(1,__3__8__)_ = 0.68 *p* > 0.05), or pGluN2A (*F*_(1,__3__8__)_ = 0.44, *p* > 0.05). No interaction effects between treatment and training were found on GluA1 (*F*_(1,38)_ = 0.15, *p* > 0.05), pGluA1 (*F*_(1,38)_ = 0.59, *p* > 0.05), GluA2/3 (*F*_(1,38)_ = 0.37, *p* > 0.05), pGluA2 (*F*_(1,38)_ = 0.93, *p* > 0.05), GluN2A (*F*_(1,38)_ = 0.33, *p* > 0.05), or p GluN2A (*F*_(1,38)_ = 0.26, *p* > 0.05).

Similarly, although no effect of anti-proBDNF antibody infusion (Figure 3E – left, two-way ANOVA, *F*_(1,__3__8__)_ = 0.45, *p* > 0.05) or interaction effect between training and treatment (*F*_(1,38)_ = 0.29, *p* > 0.05) was found, a significant effect of training (*F*_(1,38__)_ = 17.31, *p* < 0.01) on the GluN2B level was observed. Importantly, a significant effect of infusion (Figure 3E – right, two-way ANOVA, *F*_(1,38)_ = 6.26, *p* < 0.05), training (*F*_(1,38)_ = 19.93, *p* < 0.001), and interaction effect between training and treatment (*F*_(1,38)_ = 5.78, *p* < 0.05) was found in phosphorylated GluN2B(pGluN2B). Furthermore, Tukey’s test showed that the pGluN2B level of the Trained-Anti group was significantly lower than that of the Con group (*p* < 0.05). Meanwhile, there were statistical differences in pGluN2B expression between the Con and Naive groups (*p* < 0.05) and the Trained-Anti and Untrained-Anti groups (*p* < 0.05). The learning-induced pGluN2B expression was gradually weakened following MWM training (Figure 3F, two-way ANOVA, effect of time: *F*_(3,54)_ = 87.28, *p* < 0.001) and completely turned to basal level within 3 h, indicating that the upregulated activation of pGluN2B was learning-related. Furthermore, a significant downregulation by blocking proBDNF expression was detected at 1 and 2 h following the training phase (interaction effect between infusion and time: *F*_(3,54)_ = 54.59, *p* < 0.001; *post hoc*, *p* < 0.05).

Additionally, since blocking of proBDNF affected synaptic structure, it would be necessary to compare if there was difference in actin protein among groups. However, we found that the levels of β-actin were comparable (Supplementary Figure 4), indicating that the above findings were not due to differences in loading or the overall levels.

### Activation of GluN2B-Mediated Pathway Reverses Memory Consolidation Defect

To further confirm that postnatal blockage of proBDNF expression is involved in the GluN2B-mediated pathway and decipher the deteriorated effect on memory consolidation, but not the acquisition or retrieval stage, DL-TBOA, which could activate GluN2B-mediated signaling (Brancaccio et al., 2017; An and Sun, 2018), was infused into the HPC 0.5 or 2.5 h before the training phase (acquisition; Anti+TBOA0.5(a) or Anti+TBOA2.5(a)), immediately following training (consolidation; Anti+TBOA(b)), and 0.5 h before the test phase (retrieval; Anti+TBOA(c)), respectively (Figure 4A). Firstly, the escape latency of all groups, including groups that would be subgrouped to Anti+TBOA(b), Anti+TBOA(c), TBOA(a), TBOA(b), and TBOA(c) groups, did not change (Figure 4B, repeated-measures ANOVA, effect of treatment: *F*_(8,105)_ = 0.73, *p* > 0.05). In the meantime, the injection of TBOA 0.5 or 2.5 h before spatial training did not affect anti-proBDNF-infused rat’s learning ability. In the probe test, the proximity scores of the control (Con), Anti+TBOA(b), and vehicle [TBOA(a), TBOA(b), and TBOA(c)] groups were significantly shorter than that of the anti-proBDNF-infused (Anti) group (Figure 4C, one-way ANOVA: *F*_(__8__,1__15__)_ = 83.51, *p* < 0.001; *post hoc*, Con, Anti+TBOA(b), TBOA(a), TBOA(b), or TBOA(c) vs. Anti, all *p* < 0.05). The TBOA treatment before memory retrieval did not disrupt animals’ performance (*post hoc*, TBOA(c) vs. control, *p* > 0.05), indicating that the lack of a rescue effect in the Anti+TBOA(c) group was not attributed to acute effect from TBOA infusion. When the TBOA infusion was performed 2.5 h prior to the acquisition training, no statistical difference was found between the Anti+TBOA2.5(a) and Anti groups. The path proximity score of the Anti+TBOA0.5(a) group was significantly greater than those of the control, Anti+TBOA(b), TBOA(a), TBOA(b), and TBOA(c) groups (all *p* < 0.05). Meanwhile, the target quadrant (III) preference was found in the control (Figure 4D, Chi-square test, χ^2^ = 11.79, *p* < 0.001), Anti+TBOA(b) (Chi-square test, χ^2^ = 10.18, *p* < 0.01), TBOA(a) (Chi-square test, χ^2^ = 14.03, *p* < 0.001), TBOA(b) (Chi-square test, χ^2^ = 11.92, *p* < 0.001), and TBOA(c) (Chi-square test, χ^2^ = 12.25, *p* < 0.001) groups, but not the Anti (Chi-square test, χ^2^ = 0.85, *p* > 0.05), Anti+TBOA2.5(a) (Chi-square test, χ^2^ = 0.71, *p* > 0.05), and Anti+TBOA(c) (Chi-square test, χ^2^ = 0.33, *p* > 0.05) groups, indicating a reference memory disruption of the Anti, Anti+TBOA2.5(a), and Anti+TBOA(c) groups. Furthermore, although target quadrant bias was found in the Anti+TBOA0.5(a) group (Chi-square test, χ^2^ = 5.62, *p* < 0.05), no obvious difference in the time spent in the target quadrant was found between the Anti and Anti+TBOA0.5(a) groups. Therefore, the persistent effect from TBOA on the memory consolidation period could be the potential explanation of the slight memory recovery of the Anti+TBOA0.5(a) group, since the half-life of p-MeOazo-TBOA, an analog of TBOA, is longer than 3 h in 50 mm KPi buffer (pH 7.4) at 37°C (Hoorens et al., 2018). Additionally, we found that infusion of ACSF during the acquisition, consolidation, or retrieval period did not cause memory deficits (Supplementary Figure 5), and thus we could rule out an effect induced by cannula implantations. Therefore, our results indicated that the inhibition effect caused by blocking proBDNF on GluN2B-mediated pathway disrupted memory consolidation, but not acquisition ability or memory retrieval.

### Activation of GluN2B-Mediated Pathway Rescues Presynaptic Neurotransmitter Release, GluN2B-Dependent SL-LTD, and Neural Activity

Memory formation during training acted to increase NMDAR responses, which were associated with synaptic transmission and neural plasticity (Yamazaki et al., 2015; Porter and Sepulveda-Orengo, 2019). Converging evidence supported that GluN2B-NMDAR-dependent LTD was necessary to mediate spatial memory consolidation (An and Sun, 2018; Sanchez-Rodriguez et al., 2019). Importantly, the correlation between behavior and neural activity was associated with memory capacity (Yang H. et al., 2015; Feng et al., 2019). To gain insight into the mechanisms of TBOA-ameliorated memory deficits, we assessed synaptic function and neural activity, which was recorded 10 cm around the platform during the probe test.

After the last trial of the training day, synaptic transmission, PPF, and synaptic plasticity were evaluated, and the traces of the fEPSPs are presented in Supplementary Table 1. No difference was found in synaptic transmission (Figure 5A, repeated-measures ANOVA, effect of treatment: *F*_(3,36)_ = 1.17, *p* > 0.05). Blockage of proBDNF by its antibody significantly declined the PPF (Figure 5B, repeated-measures ANOVA, effect of treatment × time: *F*_(__12,144__)_ = 22.63, *p* < 0.001; *post hoc*, Anti vs. Con, 60 ms: *p* < 0.05), whereas TBOA did rescue the attenuated PPF (Anti+TBOA vs. Anti, 60 ms: *p* < 0.05). The time course of fEPSPs slopes, which were normalized to the 20-min baseline period, was depressed and reached a stable level 15 min after LFS (Figure 5C, repeated-measures ANOVA, effect of treatment: *F*_(4,45)_ = 45.77, *p* < 0.001). Post-LFS transiently enhanced depression (PTD) was measured by comparing fEPSPs that were obtained during the first minute after LFS. The fEPSPs slope of PTD from the Anti-group was obviously higher than those from the Con, Anti-TBOA, or TBOA groups (Figure 5D, one-way ANOVA, effect of treatment: *F*_(4,46)_ = 66.18, *p* < 0.001; *post hoc*, Anti vs. Con or TBOA, both *p* < 0.05). At the last 20 min of the SL-LTD recording, the mean slope of the Anti-group was markedly higher than those of both the Con and TBOA groups (Figure 5E, one-way ANOVA, effect of treatment: *F*_(4,46)_ = 37.39, *p* < 0.05; *post hoc*, Anti vs. Con or TBOA, all *p* < 0.05). As expected, TBOA could mitigate the suppressive effects of anti-proBDNF antibody on PTD (Anti+TBOA vs. Anti, *p* < 0.05) and SL-LTD (Anti+TBOA vs. Anti, *p* < 0.05). Importantly, treatment with the GluN2B antagonist Ro25-6981 completely blocked PTD (Ro25 vs. Con, Anti, TBOA, or Anti-TBOA, all *p* < 0.05) and the expression of SL-LTD (Ro25 vs. Con, TBOA, or Anti-TBOA, all *p* < 0.05). Furthermore, in a separate group of rats, proBDNF expression was blocked in adulthood (at the eighth postnatal week), but synaptic function was comparable with the vehicle group when they were tested at 12 weeks old (Supplementary Figure 6). Additionally, long-term potentiation (LTP) was induced by high-frequency stimulation (HFS, 100 pulses of 100 Hz) as published methods (An and Sun, 2018; An et al., 2019). The fEPSPs slope of LTP was assessed, but no statistical difference was observed in the fEPSPs slope between the Con and Anti groups (one-way ANOVA, effect of treatment: *F*_(1,18)_ = 0.73, *p* > 0.05; Anti: 143.67 ± 4.98; Con: 140.89 ± 4.76).

Overall, 266 units were sorted by waveform characteristics and spiking patterns (pyramidal neurons: 62 from the control (CON) group, 65 from the anti-proBDNF (Anti) group, 59 from the Anti+TBOA group, and 57 from the TBOA group; FS interneurons: 6 from the CON group, 6 from the Anti-group, 5 from the Anti+TBOA group, and 6 from the TBOA group) (Supplementary Figure 7A). Application of anti-proBDNF antibody did not affect the percentage of population (Figure 5F, Pearson χ^2^ test, *p* > 0.05). Blockage of proBDNF expression significantly decreased the firing frequency of pyramidal neurons around the targeted platform (Figure 5G, repeated-measures ANOVA, effect of treatment × time: *F*_(__3_,_36__)_ = 12.73, *p* < 0.001; *post hoc*, Anti vs. Con or TBOA, target: both *p* < 0.05), but not out of the target area. Furthermore, activation of GluN2B effectively enhanced the firing rate during memory test (Anti+TBOA vs. Anti, target: *p* < 0.05). No effect of treatment or time was found in FS interneurons (Figure 5H, effect of treatment: *F*_(3,36)_ = 0.10, *p* > 0.05). Additionally, there was no statistical difference in firing frequency of pyramidal neurons (Supplementary Figure 7B) of FS interneurons (Supplementary Figure 7C) during the baseline recording, which was conducted in rats’ home-cage.

Overall, these findings further confirm that the impaired synaptic function and neural correlates of memory consolidation contribute to the cognitive deficits induced by blocking proBDNF expression. These observations also suggest that activation of the GluN2B-mediated pathway by TBOA can be one of the key measures for rescuing the memory disability.

## Discussion

Mature BDNF has been investigated for its positive roles in regulating synaptic development and function. Although it is established that proBDNF serves diverse biological functions (Guo et al., 2016), its role in the development of spatial cognition has been debated. In the present study, multiple lines of evidence demonstrated that the expression of hippocampal proBDNF in the fourth postnatal week played a vital role in spatial memory consolidation, but not in memory acquisition or retrieval. The study uncovered three striking features of postnatal proBDNF that were not previously recognized: first, the spine density and the proportion of mature spines declined in adults following the blocking of proBDNF in the fourth postnatal week. Second, blocking postnatal proBDNF attenuated synaptic function, including PPF, PTD, and SL-LTD, which were associated with the reduction in learning-induced pGluN2B expression. Third, the activation of the GluN2B pathway by TBOA immediately following acquisition training could effectively mitigate proBDNF-mediated memory deficits and synaptic responses and elevate the memory-related activity of pyramidal neurons in the HPC.

In support of the proBDNF levels that peaked at PD24, the mBDNF level was downregulated during a transient period of NMDAR-dependent inhibition/excitation imbalance around PD28 (Zhang et al., 2018). Moreover, Orefice et al. (2013) found a similar critical period contributing to the distinct roles of somatically and dendritically synthesized mBDNF in spine shape and density. More specifically, the effect of proBDNF on spine density was not initiated at PD21 but between PD21 and PD28 during which spine pruning occurred (Orefice et al., 2016). Using mice expressing two alleles of bdnf with a HA tag to detect BDNF isoforms, Yang et al. (2014) found that the hippocampal proBDNF level was the highest at PD15, with a reduction at PD42 or later. This finding indicated that the effects of endogenous proBDNF protein would be the most robust in early postnatal development, consistent with the higher levels of p75^*NTR*^ in the HPC at the early age (Woo et al., 2005; Yang J. et al., 2009), particularly in CA1 pyramidal cell apical dendrites, postsynaptic to the Schaffer collateral axon terminals (Woo et al., 2005). They also found that the potent effects of proBDNF played a role in the development of hippocampal circuitry, which might influence hippocampal-dependent functions later in life, as demonstrated in this study. Consistent with the crucial role of BDNF in spine outgrowth (Greenberg et al., 2009; Deinhardt and Chao, 2014), our findings indicated that proBDNF was required for spine development, and the blockage of proBDNF expression resulted in spine loss. A higher proportion of thin immature spines implied the role of postnatal proBDNF in spine pruning (Guo et al., 2016; Orefice et al., 2016). Actually, thin spines are thought to be highly motile and unstable structures characteristic of immature synapses, which can be transformed into more mature and stable phenotypes during early development (Dunaevsky et al., 1999). Therefore, the impairment of spatial memory consolidation may be attributed to the decline in the mushroom spine, which is strongly associated with memory formation (Bourne and Harris, 2007). Previous studies showed that proBDNF had an effect on learning strategy (An et al., 2018) and extinction of contextual fear memory but not on learning ability (Sun et al., 2018a). Importantly, blocking postnatal proBDNF did not result in an inefficient learning strategy, indicating that the deficit in memory consolidation was driven by a less precise learning strategy. Similar to previous findings (An et al., 2018; Sun et al., 2019), proBDNF-induced memory defects were not a result of impaired locomotion, anxiety-like behavior, or motivation. The specific mechanism of spine pruning remains unclear. The synaptic transmission and presynaptic calcium ion levels play significant roles (Segal et al., 2000). The notion is supported by the diminished PPF, which has been used as a measure of changes in presynaptic Ca^2+^ dynamics and neurotransmitter release probability (Burnashev and Rozov, 2005).

QQNMDAR activation stimulates both translation of dendritic BDNF mRNA and secretion of its translation products, mainly as proBDNF, which promotes spine maturation (Orefice et al., 2016). Depending on the age of the animals, the dynamic changes in the expression of GluN1, GluN2A, and GluN2B subunit mRNAs can lead to different mixtures of NMDA receptors in the developing HPC (Sans et al., 2000; Law et al., 2003). For example, higher GluN2B expression is found in postnatal brains, but GluN2A gradually becomes more prevalent in adulthood and advanced ages (Hestrin, 1992; Monyer et al., 1992, 1994). Considering the crucial role of proBDNF-p75^*NTR*^ signaling in GluN2B-mediated spine maturation and synaptic function (Woo et al., 2005; Yang et al., 2014; Orefice et al., 2016), it is plausible that the increased expression of GluN2B subunit during postnatal weeks may be a critical mediator in proBDNF-mediated spine pruning and memory functions. The GluN2B mRNA levels peaked during the neonatal period, which was also observed in humans, with a decline to reach adult levels by 6–12 months (Law et al., 2003). BDNF mRNA levels increase approximately from 5-month infancy to adolescence and are maintained at a constant level throughout adulthood and aging (Webster et al., 2002). Interestingly, the significant increase in BDNF mRNA levels in the dorsolateral prefrontal cortex coincides with the time when the frontal cortex matures both structurally and functionally (Webb et al., 2001; Webster et al., 2002). Furthermore, the first postnatal month is characterized by an increase in the number of excitatory synapses (Steward and Falk, 1991). The activity-dependent activation of NMDA receptors can switch the effects of the proBDNF-p75^*NTR*^ pathway on synaptic activity from potentiation to depression in the developing HPC (Langlois et al., 2013). The critical period of the increases in GABAergic inhibition, which is from the fourth toward the end of the fifth postnatal weeks, is overlapped with the time of peak proBDNF expression, suggesting a transitory period of synaptic balance during development (Zhang et al., 2018). Thus, the number and efficiency of inhibitory synapses may also be regulated during the postnatal days to adjust the strength of inhibition so as to counter the increased number of excitatory synapses. Given that NMDAR-mediated signaling is essential for the effects of BDNF on dendritic development (Finsterwald et al., 2010), blocking proBDNF during the early postnatal period may induce neurotransmission impairments, further leading to spine reduction. Future experiments are required to prove this hypothesis.

Memory formation during training acts to increase AMPAR and NMDAR phosphorylation (Mizuno et al., 2003; Barki-Harrington et al., 2009; Solomonia et al., 2013). Spatial learning induces the phosphorylation of hippocampal TrkB, Fyn, and GluN2B, which are associated with memory formation (Mizuno et al., 2003). The age-related declines in GluN2B expression in the frontal cortex are related to spatial reference learning deficits (Zamzow et al., 2016). Indeed, learning-induced tyrosine 1472 allows for the enhanced binding of GluN2B with PSD95, concentrating and holding NMDAR on synaptic membranes, and increasing synaptic function (Roche et al., 2001; Barki-Harrington et al., 2009; Xu, 2011). Moreover, the expression levels of GluA1, GluN2A, and GluN2B subunits of NMDAR are altered in the insular cortex after taste learning (Barki-Harrington et al., 2009). The differences in expression and phosphorylation of AMPAR and NMDAR subunits from different studies could be attributed to the differences in the fractionation protocol of learning tasks and specific brain areas (Adaikkan and Rosenblum, 2012).

Spines are the primary site for excitatory/inhibitory inputs to neurons, and a reduced spine number and changes in morphology contribute to synaptic dysfunction. Notably, proBDNF is known to facilitate synaptic depression at hippocampal synapses by mediating presynaptic glutamate release and by regulating activation of postsynaptic glutamatergic receptors (Yang F. et al., 2009; Yang J. et al., 2009). Intriguingly, the downregulation of postnatal proBDNF levels does not affect the expression of glutamatergic receptors, but results in the suppression of learning-induced phosphorylation of the GluN2B-NMDA receptor, which has been associated with the induction of LTD (Ge et al., 2010; An and Sun, 2018). One underlying presynaptic mechanism of PTD is the rising Ca^2+^ concentration in terminal boutons (Burnashev and Rozov, 2005), disturbing the induction of long-term plasticity (Fioravante and Regehr, 2011). Furthermore, the phosphorylation of the GluN2B subunit is essential for activating a signaling cascade leading to the activation of memory-related plasticity (Zhou et al., 2007). It concurred with a previous finding that GluN2B-dependent LTD played pivotal roles in post-learning information sculpting (Dietz and Manahan-Vaughan, 2017). Other findings also indicated that memory consolidation rather than memory acquisition required the NMDAR-LTD mechanism to modify the hippocampal circuit to store fear memory (Liu et al., 2014). Previous evidence indicated that hippocampal GluN2B-dependent LTD could be induced following DL-TBOA infusions *in vitro* (Kratzer et al., 2012) and *in vivo* (Wong et al., 2007; An and Sun, 2018). In fact, DL-TBOA blocked the recycling of presynaptically released glutamate and caused accumulation of glutamate in the synaptic cleft, thus enhancing “spillover” and increasing the likelihood of extrasynaptic GluN2B-NMDA receptor activation (Massey et al., 2004; Yang et al., 2005). Additionally, the inhibitory effect of Ro25 on the induction of SL-LTD suggested that our findings were due to specific enhancement of GluN2B-dependent SL-LTD. Furthermore, a significant influence of postnatal proBDNF on HPC neuronal activity during memory formation and the involvement of GluN2B-mediated signaling in the memory consolidation process were found in the present study. Previous studies found that proBDNF-mediated p75^*NTR*^ activation was responsible for controlling the performance in spatial memory tests and HPC excitability (Woo et al., 2005; Barrett et al., 2010). Our findings had some overlap with the evidence that mBDNF reduced action potential firing of FS cells in the hippocampal dentate gyrus, whereas proBDNF had no effect (Holm et al., 2009). Consistently, the training-induced increase in proBDNF expression promoted the firing rate of pyramidal neurons but not FS interneurons (An et al., 2018). Therefore, our findings extended the understanding of the effects of proBDNF on spatial memory function, which were mostly attributed to its actions on the learning-induced phosphorylation of GluN2B subunits and GluN2B-dependent neural function.

Through mediating C-terminal ubiquitination, TBOA can substantially enhance polyubiquitination of the GluA1 receptors (Jarzylo and Man, 2012). Presynaptically, an increase in glutamate concentrations in the early phase in the active synapse induced by low concentrations of DL-TBOA can be masked by AMPAR desensitization (Takayasu et al., 2004). Furthermore, the enhancement of the sodium ion current evoked by TBOA is attributed to its interaction with sodium ion carrier proteins, such as Na,K-ATPase (Bozzo and Chatton, 2010), which is co-localized with NMDA receptors and forms a function complex either by interacting directly or through some intermediate proteins (Akkuratov et al., 2015). Therefore, the rescuing effects of TBOA on GluN2B-NMDARs may be also involved in its effects on the activation of other glutamate receptors.

Spine maturation and pruning depend on neuronal activity and are required to refine neuronal connections in the developing brain (Segal et al., 2000; Bourne and Harris, 2007). Previous observations show that the long 3’UTR Bdnf mRNA, which is transported to dendrites for local translation (An et al., 2008), is essential for head enlargement and pruning of dendritic spines *in vivo* and *in vitro* (An et al., 2008; Kaneko et al., 2012; Orefice et al., 2013). For example, mice lacking long 3′UTR Bdnf mRNA display thinner and denser spines on the dendrites of CA1 pyramidal neurons in the HPC and L2/3 pyramidal neurons in the visual cortex (An et al., 2008; Kaneko et al., 2012). Furthermore, knocking down long 3′UTR Bdnf mRNA or blocking the transport of long 3′UTR Bdnf mRNA to dendrites inhibits spine maturation and pruning, whereas overexpressing long 3′UTR Bdnf mRNA enhances spine maturation and pruning in cultured hippocampal neurons (Orefice et al., 2013). Interestingly, the translation product of long 3′UTR Bdnf mRNA is mainly secreted as precursor BDNF. The overexpression of dendritic proBDNF alone or dendritic proBDNF plus 3’UTR Bdnf mRNA caused a significant increase in spine head width. More importantly, granule cells in p75^*NTR*^ knockout mice had significantly smaller spine heads at both PD21 and PD28. These findings indicated that dendritically synthesized proBDNF from 3’UTR Bdnf mRNA promoted spine pruning and maturation *via* p75^*NTR*^ (Orefice et al., 2016). The mechanisms by which proBDNF coincidently mediates the pruning and maturation of dendritic spines are unclear. However, the materials from eliminated spines may be recycled to activate spines, thus facilitating their growth. However, the hypotheses need further investigation.

The present results did not replicate the findings of a previous study, which showed increased spine density following spatial maze training and a correlation between spine density and behavioral performance (Mahmmoud et al., 2015; Dillingham et al., 2019). Actually, hippocampal dendritic spines are temporally dynamic structures, and as such, the time at which they are assessed may be a critical factor. A previous study found changes in CA1 spine clustering, but no change in density, 6 days after water-maze training (Rusakov et al., 1997). More detailed information on the time course of CA1 spine formation and turnover can be acquired from slice studies. For instance, initial plasticity, including spinogenesis along the dendritic shaft of CA1 neurons, following stimulation was designed to mimic long-term potentiation (Bourne and Harris, 2011). However, no overall change in spine density was observed 2 h after stimulation, suggesting a redistribution of spines and a balance between the loss and gain of spines (Bourne and Harris, 2011). Functional entorhinal cortex coupled with CA1 activity became more direct with additional training, thus producing a trisynaptic circuit bypass (Poirier et al., 2008), hence suggesting that the stage of learning was another critical factor in the eight-trial training-induced structural changes. One more possibility was that the typical light microscopy used in the current and previous studies did not have sufficient spatial resolution to properly resolve the distinguishing features of spines (Harland et al., 2014; Wartman and Holahan, 2014). For example, Tonnesen et al. used super-resolution stimulated emission depletion imaging and found only few stubby spines (Tonnesen et al., 2014). Future studies, using a continuous spectrum, as suggested elsewhere (Yuste and Majewska, 2001; Arellano et al., 2007; Gipson and Olive, 2017), may provide more detailed information.

Some studies indicated an increase in proBDNF in the aged mouse HPC (Buhusi et al., 2017), whereas other studies showed that the aging-related accumulation of proBDNF did not occur (Michalski and Fahnestock, 2003; Silhol et al., 2007). The adverse effects of proBDNF accumulation over time in aged rodents would affect neuronal morphology and spine density, leading to synaptic and behavioral deficits (Perovic et al., 2013; Buhusi et al., 2017). Consistent with the NMDA-dependent switch of proBDNF actions on developing synapses (Langlois et al., 2013), our findings might extend these findings and indicate a bidirectional regulation of proBDNF in distinct developmental stages. Interestingly, spatial training increased proBDNF metabolism in both young and aged rats (Silhol et al., 2007). Studies performed on experimental/transgenic animals indicated that proBDNF tended to facilitate mature spines pruning. A recent study demonstrated that the effects of BDNF on the dendritic architecture of the hippocampal neurons were dependent on the neuron’s maturation stage (Kellner et al., 2014). Furthermore, the interaction between compensatory mechanisms and gene environment may ultimately determine the lack of the effects of BDNF on the regulation of spine maturation and pruning (Orefice et al., 2016). Hence, it is important to note that blocking proBDNF expression by its antibody during the postnatal period, rather than gene mutations, should be an essential approach to provide direct evidence for its effects on brain function. Furthermore, the mechanism by which proBDNF exerts its effects, other than it being related to the GluN2B subunit, still needs further investigation, especially given that the effects were found after the developmental GluN2B to GluN2A shift. The estrogenic regulation of BDNF signaling is likely sex specific (Chan and Ye, 2017; Wei et al., 2017). Intriguingly, the inherent organization of the HPC in terms of hormonal responses is programmed early in life (Hill et al., 2012; Kight and McCarthy, 2017). In ovariectomized female rats, BDNF protein and mossy fiber synaptic function decreased, whereas orchidectomy led to what would seem to be the opposite effect in male rats (Scharfman and MacLusky, 2014). Presumably, the neonatal surge in hormone and BDNF levels, which accompany the sex differences in brain development, leads to a circuitry upon which adult BDNF levels exert a varying influence. Moreover, the sexual differences in neuronal signaling, especially those induced by BDNF, are observed in an early stage (Chan and Ye, 2017). Therefore, further experiments involving the sex-specific effects of proBDNF may provide more information.

In this study, we demonstrated that the blockage of proBDNF expression during the fourth postnatal week disrupted spatial memory consolidation by structurally reducing the ratio of mature spines and functionally suppressing synaptic function and neural activity. The learning-induced phosphorylation of GluN2B subunits is likely an important mechanism in inducing LTD and promoting neural correlate with the memory consolidation process. Taken together, our findings are important for obtaining a unifying concept of the biological roles of proBDNF in cognitive and neural functions.

## Data Availability Statement

The raw data supporting the conclusions of this article will be made available by the authors, without undue reservation.

## Ethics Statement

The animal study was reviewed and approved by the Ethics Committee on the Care and Use of Animals Committee of Guizhou University of Traditional Chinese Medicine.

## Author Contributions

WS, YY, DT, and LA conceived and designed the experiments. WS, HC, and XL performed the experiments. WS, HC, XL, and LA analyzed the data. WS, YY, and LA wrote the manuscript. All authors contributed to the article and approved the submitted version.

## Conflict of Interest

The authors declare that the research was conducted in the absence of any commercial or financial relationships that could be construed as a potential conflict of interest.
