# Supplementary material for: Requirements of Postnatal proBDNF in the Hippocampus for Spatial Memory Consolidation and Neural Function
Source: Front Cell Dev Biol. 2021 Jul 15;9:678182. doi: 10.3389/fcell.2021.678182 (PMC8319730; doi:10.3389/fcell.2021.678182)
Supplement: Supplementary file 1 [file Data_Sheet_1.pdf]

Supplementary information

SI Methods

Silver staining

Silver staining was used to observe the morphological alterations. Samples were fixed in 4% paraformaldehyde for 30 min and rinsed twice in PBS for 5 min and once with distilled water. Then the slides were immersed in pre-warmed (37 ℃) 10% filtered silver nitrate and stained for 30 min. Six milliliters of concentrated ammonium hydroxide was added to the flask containing the silver nitrate solution, and the solution was cleared. Ammoniacal silver was poured onto the slides, which were stained for 15 min at 37 ℃, then exchanged for a 1% ammonium hydroxide solution for 3 min and then returning the ammoniacal silver to a flask and adding 25 drops of fresh developing solution. The slides were placed in this solution for 5 min and then transferred to 1% ammonium hydroxide solution for 3 min and to 5% sodium thiosulfate solution for 5 min. The slides were rinsed for three times in distilled water, and 5 min for each time. Finally, they were dehydrated and cleared with 95% ethyl alcohol, absolute alcohol, and xylene. The slides were mounted with a resinous medium. Silver positively stained neurons were quantified using threshold analysis to evaluate neurodegeneration. The threshold value was set to consistently detect maximal positive staining of silver and the quantification was performed by the NIH Image program (http://rsb.info.nih.gov/nih-image/)

T-maze task

Training was conducted in a black Plexiglas cross-maze consisting of 2 start arms and 2 test (reward) arms, which were diverged at a 90° angle from each other. Only one start arm was used during the training days, the other start arm was blocked by removable black Plexiglas barrier. Rat was allowed to visit all available maze arms until it obtained reward within 3 min. After reward consumption, the experimenter placed the rat in the inter-trial box for 10-20 s. After cleaning all test arms and re-baiting the same arm. For each individual animal, the reward location was fixed,

however, reward location pseudorandomly between subjects and treatments during each training day. Rats were subjected to six trials per day until the choice accuracy was 90% or above. On the following day, they were subjected to a probe trial to assess the learning strategy used during training stage. During the probe trial, the animals started from the opposite start arm while the original start arm was blocked. Rats were rewarded whatever the choice they made in probe test. The same maze and environment were used, thereby insuring common sensory, behavioural, and motivational experiences. The probe trial had two possible outcomes: (1) rats using a place strategy would visit the arm that was baited during training or (2) rats using a response strategy would make the same turn as they had done during training and would visit the other arm.

SI Figures and Results

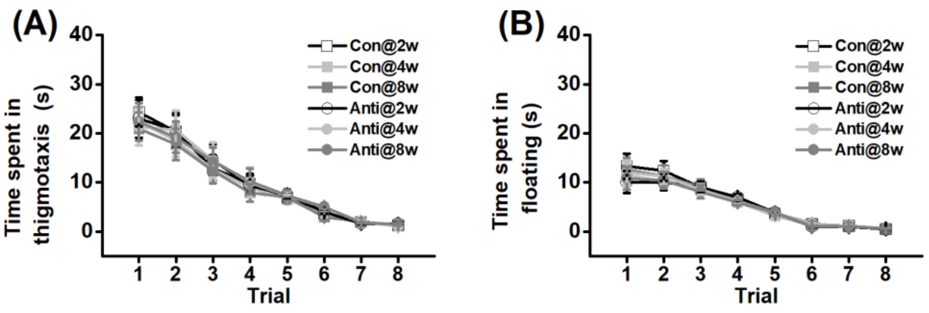

Fig. S1. The mean time spent in thigmotaxis and floating during the spatial acquisition of the MWM test.

As our previous study (*1*), to determine whether the differences in performance were attributable to cognitive or non-cognitive factors, the time spent in thigmotaxis **(A)**, which was thought to be a measure of anxiety like behaviour, and the time spent in floating **(B)**, which was considered as non-mnemonic behaviour, were measured. No statistical difference was found in thigmotaxis (repeated-measures ANOVA, interaction effect between trial and treatment: $F_{(35,\ 348)}$=1.21, P>0.05) or floating (repeated-measures ANOVA, interaction effect between trial and treatment: $F_{(35,\ 348)}$=0.97, P>0.05) behaviours among groups. n=5 for Con@2w group, n=5 for Con@8w group, n=6 for Anti@8w group and n=16 for other each group.

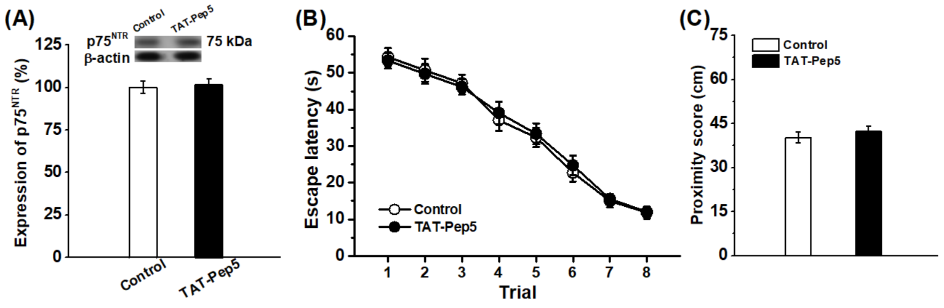

Fig. S2. Inactivation of p75^{NTR} expression during postnatal period does not affect its expression and behavioural performance.

To rule out changes in p75^{NTR} function as a result of blocking proBDNF expression, rats (TAT-pep5 group) were infused with TAT-pep5 into the CA1 region during the fourth postnatal week and tested during the eight-week old. Meanwhile, control rats

were infused with ACSF as the vehicle. **(A)** The expression of $p75^{NTR}$ in the hippocampal CA1 region was comparable between control and TAT-pep5 groups (T-test, t_{12}=0.0, P>0.05). The escape latency (two-way ANOVA, effect of treatment, $F_{(1,\ 12)}$=0.17, P>0.05; interaction effect between trial and treatment: $F_{(7,\ 84)}$=0.08, P>0.05) **(B)** and the proximity score (T-test, t_{12}=0.1, P>0.05) **(C)** in the Morris water maze task were not affected by infusion of TAT-pep5 during the fourth postnatal week. n=6 for control group, n=8 for TAT-pep5 group. These findings indicate blocking proBDNF expression but not affecting the p75 receptor expression or function by the infusion of anti-proBDNF antibody induces behavioural deficits of the adults.

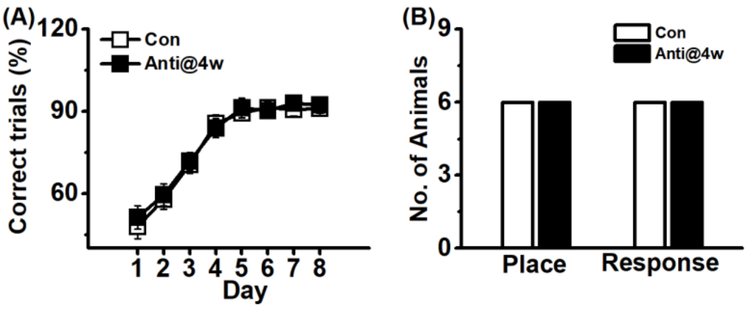

Fig. S3. Blocking proBDNF expression during the 4th postnatal week does not induce the learning strategy preference.

To test whether blocking proBDNF expression during the fourth postnatal week induced learning strategy bias, rats that were infused with anti-proBDNF antibody (Anti@4w) and ACSF (Con) respectively were tested in the T-maze task. **(A)** No significant difference was found between two groups (two-way ANOVA, effect of treatment: $F_{(1,\ 10)}$=0.21, P>0.05; interaction effect between trial and treatment: $F_{(7,\ 84)}$=0.11, P>0.05). Therefore, infusion anti-proBDNF did not affect strategy learning process. **(B)** The number of rats used place or response strategy during the probe test. Anti@4w group did not show a preference for place or response strategy in the probe trial (P<0.01, binomial test), which was conducted one day following the strategy learning. Statistical comparison indicated that there was no statistical difference in the use of learning strategy between Anti@4w and Con groups (P<0.01, Pearson χ^2 test).

n=12 for each group.

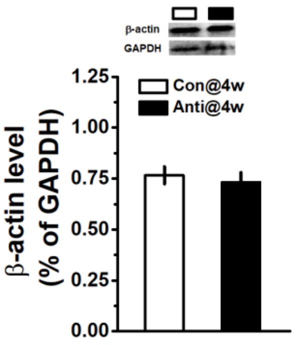

Fig. S4. The expression of β–actin in the Con@4w and Anti@4w groups.

Mouse anti-β-actin (1:20,000; Cat#A5316, Sigma) and mouse anti-GAPDH (1:20,000; Cat#MAB374, Chemicon) and GAPDH were applied as primary antibodies. The GAPDH was used as an internal control. There was no statistical difference between two groups (T-test, t_{10}=0.0. P>0.05), indicating that the findings in this study were not due to differences in loading or the overall levels.

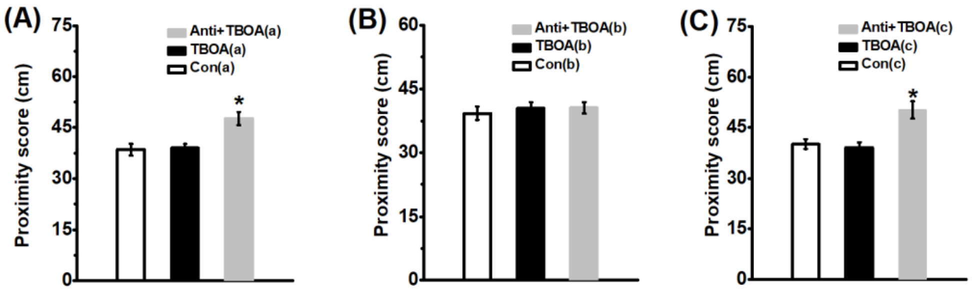

Fig. S5. The potential effects induced by cannula implantations.

To rule out the potential effects of the stress induced by cannula implantations, the same volume of ACSF were bilaterally infused 0.5 h before spatial training (Con(a)), immediately after training stage (Con(b)) and 0.5 h before probe test (Con(c)). Compared with each matched TBOA groups, the swim proximity score of Con(a), Con(b), or Con(c) was not significantly affected. Additionally, the proximity score of Con(a) and Con(c) was statistical lower than its matched Anti+TBOA group. Therefore, the findings in the current study were not involved in the stress effects induced by the cannula implantations. *P<0.05, vs. other groups. *n*=4 for Con(a), *n*=4 for TBOA (a), *n*=10 for Anti+TBOA (a), *n*=20 for Con(b), *n*=20 for Anti+TBOA(b),

n=20 for TBOA(b) group, n=4 for Con(c), n=5 for TBOA(c) group and n=10 for Anti+TBOA(c).

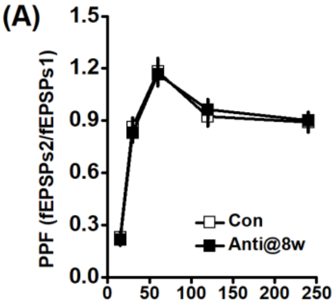

Fig. S6. Blocking proBDNF expression during the 8th postnatal week does not disrupt synaptic function.

To further verify that the 4th postnatal week is the critical period for the profound effects of proBDNF expression on synaptic function, we tested whether blocking proBDNF expression had a similar effect even on older rats. Anti-proBDNF antibody was bilaterally infused into the hippocampus of 8-week-old adult rats, synaptic function was tested about four weeks later. The fEPSPs slope of **(A)** Paired-pulse facilitation (PPF; two-way ANOVA, effect of treatment: $F_{(1,\ 11)}$=0.24, P>0.05; interaction effect between trial and treatment: $F_{(4,\ 44)}$=0.16, P>0.05), **(B)** Post-LFS transiently enhanced depression (T-test, t_{11}=0.0, P>0.05), **(C)** Long-term depression (LTD; T-test, t_{11}=0.0, P>0.05) were comparable between two groups. n=6 for control group, n=7 for Anti@8w group. Therefore, blocking proBDNF in adulthood does not result in synaptic impairments and further confirm the specific roles of proBDNF during the 4th postnatal week in spatial memory and synaptic function.

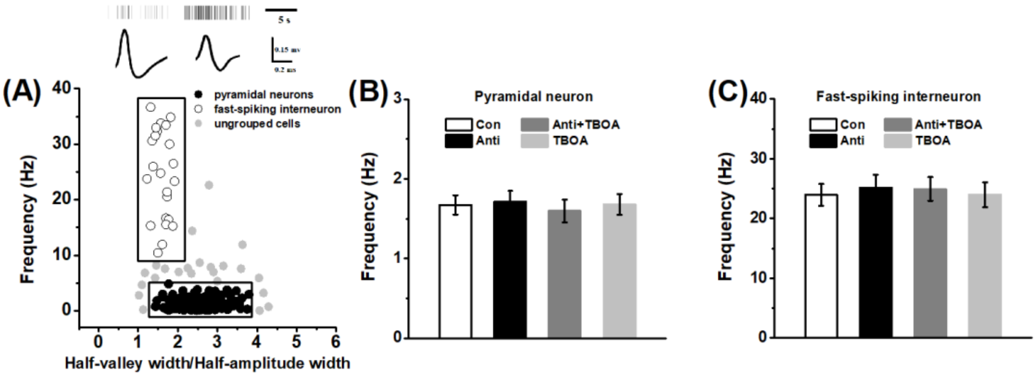

Fig. S7 Blocking proBDNF expression during the 8th postnatal week does not disrupt synaptic function.

(A) Distribution of average firing rate and half-valley to half-peak ratio of hippocampal neurons. The firing frequency of pyramidal neurons positively correlated with the spike width (Pearson's r=0.21; P<0.01). Therefore, the wide spikes of pyramidal neurons tended to have large half-valley to half-peak ratio. On the other side, a significant negative correlation between the mean frequency and the ratio of half-valley to half-peak of the spike waveform was found (Pearson's r= -0.39; P<0.001). So the high frequency fast-spiking interneurons tended to have spike waveforms with relatively small half-valley to half-peak ratio. Neurons were therefore separated into two groups as indicated by rectangular lines. The top inset represented the raster plot of the spike times and the waveform of pyramidal and FS cells, respectively. No statistical difference in the frequency of pyramidal neurons (one-way ANOVA, effect of treatment: $F_{(3, 239)}$=0.22, P>0.05) **(B)** and fast-spiking interneurons (one-way ANOVA, effect of treatment: $F_{(3, 19)}$=0.29, P>0.05) **(C)** was found during the baseline recording, which was conducted in rats' home-cage.

Table S1 The traces of the fEPSPs during the I/O, PPF and LTD recordings.

	Group				
	Con	Anti	Anti+TBOA	TBOA	Ro25
I/O fEPSPs	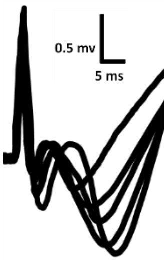	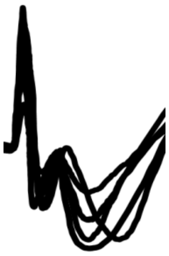	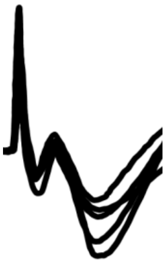	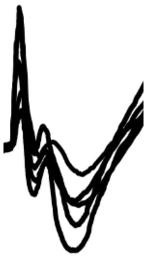	N/A
PPF at the inter-pulse intervals of 60 ms	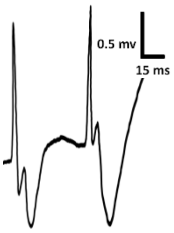	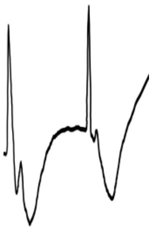	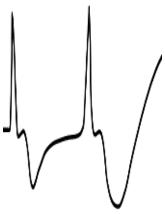	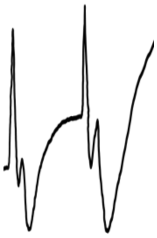	N/A
fEPSPs during LTD recordings	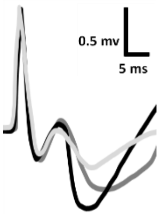	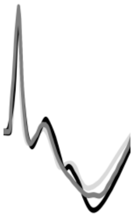	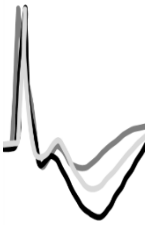	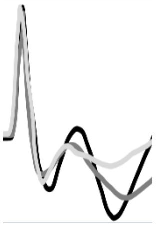	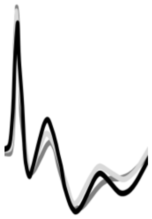

Note: In the LTD recording, the light gray traces indicated the traces of the fEPSPS of the post-LFS transiently enhanced depression. The dark gray ones indicated the traces of the fEPSPs, which were taken at 41 min after LFS. The black ones indicated the traces of fEPSPs of baseline.

References

1. An, L., and Zhang, T. (2013) Spatial cognition and sexually dimorphic synaptic plasticity balance impairment in rats with chronic prenatal ethanol exposure, *Behavioural Brain Research 256*, 564-574.
